#           Toxin-Specific Antibodies for the Treatment of *Clostridium difficile*: Current Status and Future Perspectives ^†^

**DOI:** 10.3390/toxins2050998

**Published:** 2010-05-07

**Authors:** Greg Hussack, Jamshid Tanha

**Affiliations:** Institute for Biological Sciences, National Research Council of Canada, 100 Sussex Drive, Ottawa, Ontario, K1A 0R6, Canada; Email: Greg.Hussack@nrc-cnrc.gc.ca

**Keywords:** *Clostridium difficile*, *Clostridium difficile*-associated disease, toxin, antibody, single-domain antibody, neutralization, therapy

## Abstract

Therapeutic agents targeting bacterial virulence factors are gaining interest as non-antibiotic alternatives for the treatment of infectious diseases. *Clostridium difficile* is a Gram-positive pathogen that produces two primary virulence factors, enterotoxins A and B (TcdA and TcdB), which are responsible for *Clostridium difficile*-associated disease (CDAD) and are targets for CDAD therapy. Antibodies specific for TcdA and TcdB have been shown to effectively treat CDAD and prevent disease relapse in animal models and in humans. This review summarizes the various toxin-specific antibody formats and strategies under development, and discusses future directions for CDAD immunotherapy, including the use of engineered antibody fragments with robust biophysical properties for systemic and oral delivery.

## 1. Introduction

*Clostridium difficile* is a Gram-positive, endospore-forming, anaerobic, gastrointestinal pathogen that is a leading cause of nosocomial infections in developed nations. The bacterium is transmitted by the fecal-oral route and can readily colonize persons with suppressed microflora as a result of antibiotic usage. The symptoms of *C. difficile* infection range from mild cases of diarrhea to fatal pseudomembranous colitis and are collectively known as *C. difficile*-associated disease (CDAD) [1,2,3]. The recent emergence of hypervirulent and antibiotic-resistant *C. difficile* strains with increased morbidity, mortality and recurrence rates [4,5] have warranted the development of novel, non-antibiotic based treatment regimes. *C. difficile* exerts its pathological effects by colonizing luminal surfaces of the colon and secreting two high-molecular weight exotoxins, toxin A (TcdA) and toxin B (TcdB). With their causative role in CDAD firmly established [6,7,8,9], these two virulence factors have been identified as targets for therapeutic intervention. With the continued rise of antibiotic resistance, the development of novel, non-antibiotic agents, which target bacterial virulence factors and reduce the selection pressure normally placed upon pathogens by antibiotics, are highly desirable [10,11,12]. These agents, such as antibodies, may also be useful to control the recurrence of infection after antibiotic treatment has been terminated.

## 2. Toxin Structure and Function

Similar to other members of the large clostridial family of toxins, TcdA and TcdB target the Rho/Ras superfamily of GTPases by irreversible modification through glucosylation [13,14]. Since GTPases are key cellular regulatory proteins, their permanent inactivation causes disruptions in essential cell signaling pathways that are critical for transcriptional regulation, apoptosis, cytoskeleton integrity and eventually colonic epithelial cell barrier function [15,16].

Before *C. difficile* can exert a physiological effect on a host, the pathogen must colonize the host. It is believed that *C. difficile* spores are consumed orally and travel to the large intestine where they flourish in environments lacking competition from normal gut microflora. Surface layer proteins (SLPs), which decorate the pathogen’s surface, are involved in adherence to the human intestinal epithelium and are thought to be a critical step in gut colonization [17]. Quorum sensing molecules have been shown to play an important role in transcriptional regulation of toxin production [18] suggesting toxin production is a cell-density dependent process. Whether *C. difficile* toxin production and secretion occurs during or after colonization of the host is unknown.

**Figure 1:**
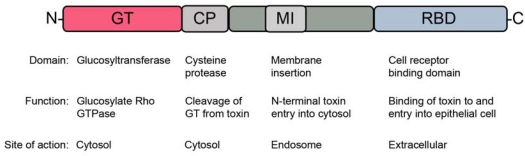
Schematic representation of *Clostridium difficile* toxin A and B. For illustration purposes, only one toxin is shown. Toxin A (TcdA, 308 kDa) and toxin B (TcdB, 269 kDa) are each composed of four domains, which perform distinct functions. The schematic illustrates each domain, their function, and site of action. GT = glucosyltransferase domain, CP = cysteine protease domain, MI = hydrophobic membrane insertion domain, RBD = cell-receptor binding domain.

**Figure 2:**
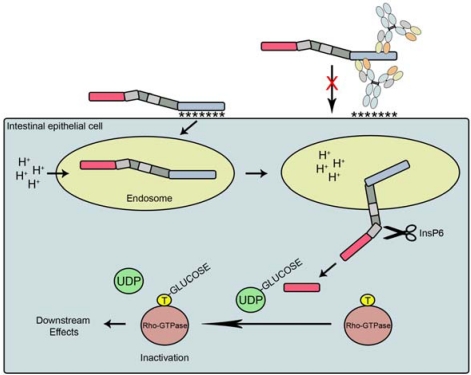
Schematic illustration of *Clostridium difficile* toxin mechanism of action. TcdA or TcdB first binds the surface of epithelial cells via the RBD region of the toxin, promoting receptor-mediated endocytosis. Acidification of the endosome-encapsulated toxin promotes a conformational change in which the N-terminal region of the toxin is extended through the endosomal membrane into the cytoplasm. Cellular inositol hexakisphosphate (InsP6) promotes cleavage at the start of the CP domain, releasing the GT domain into the cytoplasm. The GT domain transfers a glucose moiety from UDP-glucose to a threonine (T) residue on Rho-GTPase, trapping the signaling enzyme in an inactive conformation. Targeting the RBD domain with antibodies and antibody fragments may block toxin binding to cell-surface receptors or prevent internalization of the toxin.

TcdA and TcdB are single-polypeptide chain, high-molecular weight exotoxins (308 kDa and 269 kDa, respectively) organized into multi-domain structures [13,19]. The genes encoding TcdA and TcdB, *tcdA* and *tcdB*, are located in the 19.6 kb *C. difficile* pathogenicity locus (PaLoc) and are positively regulated at the protein level by TcdR [14]. Like other members of the large clostridial toxin family, TcdA and TcdB are organized as modular domains with each domain performing a distinct function (Figure 1). The C-terminal region of TcdA/B is responsible for toxin binding to the surface of epithelial cells possibly via multi-valent interactions with putative cell-surface carbohydrate receptors [20,21]. Structural studies of this cell receptor binding domain (RBD) from TcdA and TcdB revealed a β-solenoid fold [19,22] with seven carbohydrate binding sites identified for receptor binding in TcdA [21,22]. While the C-terminal region of TcdA has been shown to bind various oligosaccharides, including the trisaccharide α-Gal-(1,3)-β-Gal-(1,4)-β-GlcNac [23], the native human ligand has not been positively identified. The TcdB host cell receptor also remains unknown. Binding of TcdA/B via the RBD to epithelial cells induces receptor-mediated endocytosis, permitting entry of the endosome-encapsulated toxin into the cytoplasm (Figure 2). Once internalized, the toxins require an acidic endosome for transport to the cytosol. A decrease in endosomal pH is thought to induce a conformational change, resulting in exposure of the hydrophobic membrane insertion (MI) domain and insertion of the N-terminus (catalytic domain and cysteine protease domain) into and through the endosomal membrane via pore formation [13]. Recently, Reineke *et al.* [24] showed inositol hexakisphosphate (InsP6) from the host cell induces the autocatalytic cleavage of the *N*-terminal region at the cysteine protease (CP) site, freeing the N-terminal glucosyltransferase (GT) domain into the cytosol while the remaining portions of the toxin is left in the endosome. This finding was later supported by evidence from Egerer *et al.* [25]. Upon cleavage, the GT domain is capable of transferring glucose residues from UDP-glucose to Rho-GTPases [26], locking the important cell signaling mechanism in an inactive conformation. Inhibition of Rho-GTPases causes a series of cascading effects, including dysregulation of actin cytoskeleton and tight junction integrity. Collectively, these events lead to increased membrane permeability and loss of barrier function [27], diarrhea, inflammation, and a massive influx of neutrophils and other members of the innate immune response [2]. 

## 3. Treatment of *Clostridium difficile*-Associated Disease

The most common treatment for *C. difficile* infection currently involves discontinuing the original antibiotic in use at the time of diagnosis followed by administration of vancomycin or metronidazole antibiotics. However, resistant strains to both antibiotics have been reported [28,29]. In addition, increased CDAD recurrence rates and the prominence of hypervirulent strains over expressing TcdA and TcdB [4,5] highlight the need for novel approaches to treatment. There are several strategies under development for the treatment of CDAD (Table 1), including: various antibiotics, replenishment of patient microflora with oral probiotic therapy or fecal-transplantation therapy, development of toxin binding resins and polymers, vaccines, and toxin-specific antibodies and recombinant antibody fragments [2,30,31,32,33]. In this review, we focus on efforts to develop anti-TcdA/B antibodies for CDAD immunotherapy, report the successes and failures, and describe the challenges that lie ahead.

**Table 1:** Therapeutic strategies under development for the treatment of CDAD.

**Type**	**Description**	**Reference**
Antibiotic	Nitazoxanide	**[34]**
	Rifaximin	**[35]**
	Ramoplanin	**[36]**
	Difimicin	**[37]**
Probiotic	*Saccharomyces boulardii*	**[38]**
	*Lactobacillus spp.*	**[39]**
Fecal transplantation	Stool replacement therapy	**[40]**
Toxin binding agents	Cholestyramine	**[41]**
	Tolevamer	**[42]**
Vaccine	Toxoid-based	**[43]**
	SLP-based	**[44]**
	DNA-based	**[45]**
Antibodies	IgG, IgA, IgY, polyclonal	See Table 2 and Table 3
	scFv	**[46]**
	sdAb	**[47]**

## 4. Toxin-Specific Antibodies

The field of antibody engineering has rapidly expanded over the past few decades, proving itself as a source for high-affinity, high-specificity, protein-based binding reagents for a myriad of applications [48]. From polyclonal antibody production in animals, to hybridoma cell culture of IgG antibodies, to the rational design of high-affinity antibodies and antibody fragments via display techniques and site-directed mutagenesis, antibodies have been produced by numerous methods and against countless targets of therapeutic importance. Of the FDA-approved therapeutic antibodies on the market most are for the treatment of cancer and autoimmune disorders, although numerous antibodies targeting the virulence factors of disease-causing bacteria are in development and in clinical trials.

With respect to *C. difficile*, administering toxin-neutralizing antibodies for CDAD therapy is supported by numerous studies which have shown that patients with low anti-toxin IgG titers are more likely to experience severe effects from *C. difficile* infection and are more likely to develop recurrent rounds of CDAD. 

### 4.1. Role of antibodies in CDAD

Persons infected with *C. difficile* experience a broad-spectrum of symptoms, ranging from asymptomatic carriage to life-threatening pseudomembraneous colitis. The reasons for such varied symptoms, or lack thereof, is not fully understood. It is believed that patients who experience mild cases of CDAD tend to possess high anti-toxin A IgG serum titers [49,50,51]. Conversely, patients susceptible to relapsing *C. difficile* infection have demonstrated low anti-TcdA Ig titers, specifically IgM, IgG2 and IgG3 isotypes [49,52,53]. TcdA-neutralizing secretory IgA (sIgA) antibodies are also thought to play a role in regulating CDAD severity in the colonic mucosa [54,55]. Furthermore, many individuals develop anti-toxin A/B antibodies (*i.e.*, IgG, IgA) in the serum [51,56] and stool after a symptomatic CDAD infection [57]. The importance of anti-toxin Abs in regulating CDAD severity and relapse is highlight by the number of experimental vaccines under development. For example, toxoid-based vaccines have protected animals against *C. difficile* challenge [57]. Others have shown antibody-mediated protection can be transferred from adult hamsters to offspring through milk [8,58]. Therefore, the introduction of anti-toxin antibodies to patients suffering from severe *C. difficile* infection may be a useful approach to treat severe CDAD or reduce the incidence of recurrent CDAD infection.

### 4.2. Experimental animal studies

**Figure 3:**
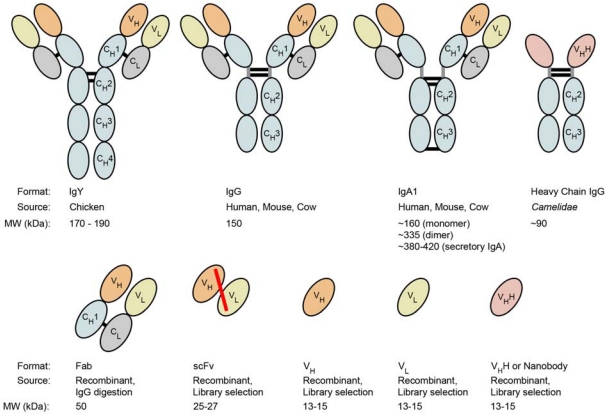
Various antibody formats for anti-toxin therapy. Traditional antibody formats (*i.e.*, IgY, IgG, IgA) targeting *C. difficile* toxins have been produced primarily from immunized animals. Smaller recombinant antibody binding fragments (*i.e.*, Fab, scFv, V_L_, V_H_, V_H_H) produced from *in vitro* selection procedures may be useful agents to explore for CDAD immunotherapy. Of these recombinant fragments, single-domain antibodies (*i.e.*, V_H_H) from *Camelidae* heavy-chain IgGs possess inherent thermal and protease stability and have been shown to bind cryptic epitopes or pockets on proteins that cannot be accessed by traditional antibodies. As such, these single-domain antibodies may be potent toxin neutralizers and promising therapeutic agents for CDAD immunotherapy. Black bars represent disulfide bonds, grey bars represent hinge regions, and the red bar represents a synthetic linker. Some Igs have more than two inter-heavy chain disulfide linkages.

Over the past 30 years, a number of antibodies (Abs) have been isolated against *C. difficile* toxins and their efficacy evaluated in various animal models (Figure 3; Table 2). Some of the earliest evidence that anti-toxin antibodies may be useful agents for *C. difficile* therapy was demonstrated by Allo *et al.* [59] who isolated *C. sordellii* toxin-specific polyclonal Abs and found intraperitoneal (I.P.) injection of these Abs into hamsters prevented clindamycin-induced *C. difficile*-associated colitis. The earliest animal study involving monoclonal antibodies (mAbs) specific for TcdA and TcdB was performed by Lyerly *et al.* [60]. This group demonstrated that pre-mixing anti-TcdA mAb PCG-4 with TcdA and orally administering the mixture completely protected hamsters from fatal doses of TcdA. However, administration of G-2 IgG, an antibody which cross-reacted with both toxins, failed to protect hamsters against oral TcdA challenge and was not capable of TcdB neutralizing *in vitro*. Elsewhere, Kamiya *et al.* [61] isolated a panel of nine TcdA-specific mAbs from hybridoma cell lines, but found none were capable of preventing mouse lethality upon I.P. co-injection of TcdA and mAb. Corthier *et al.* [62] later isolated three TcdA-specific mAbs (A9, 141-2, and C11) and found the antibodies to completely protect mice when injected intravenously four days prior to *C. difficile* challenge. This panel of mAbs was not tested in *C. difficile* post-challenge treatment models however. Interestingly, these three mAbs and PCG-4 produced by Lyerly *et al.* [60] were shown to recognize the C-terminal cell-receptor binding domain region of TcdA, indicating the antibodies may have blocked toxin-cell contacts or prevented internalization of the toxin (Figure 2).

**Table 2:** Animal studies involving *C. difficile* toxin-specific antibodies.

**Antibody**	**Specificity**	**Immunogen**	**Antibody Source**	**Animal Model**	**Challenge Type**	**Ab Administration Route **	**Treatment Type**	**Outcome**	**Ref.**
PCG-4 IgG	TcdA	Culture filtrate	Mouse	Hamster	Oral TcdA administration	Oral	Ab + TcdA co-administered	Protection	**[60]**
G-2 IgG	TcdA and TcdB	Toxoid B	Mouse hybridoma	Hamster	Oral TcdA administration	Oral	Ab + TcdA co-administered	No protection	**[60]**
37B5 IgG	TcdA	Toxoid A	Hybridoma	Mouse	I.P. TcdA administration	I.P.	Ab + TcdA co-administered	No protection	**[61]**
A9, 141-2, C11 IgGs	TcdA	Toxoid A	Mouse	Mouse	Oral *C. difficile*^1^	I.V.	Prophylactic	Protection	**[62]**
Bovine Ig	TcdA and TcdB	Culture filtrate	Cow colostrum	Hamster	Oral *C. difficile* (10^8^ cells)	Oral	Prophylactic	Protection	**[63]**
Bovine Ig	TcdA and TcdB	Culture filtrate	Cow colostrum	Rat	CD filtrate into ileum ^2^	Ileum injection ^2^	Ab + toxin co-injected ^2^	Protection	**[64]**
Anti-TcdA Bovine Ig	TcdA	Toxoid A	Cow colostrum	Rat	CD filtrate into ileum ^2^	Ileum injection ^2^	Ab + toxin co-injected ^2^	Protection	**[64]**
Anti-TcdA IgY	TcdA	rTcdA fragment	Chicken	Hamster	Oral *C. difficile* (10^4^ cells)	Oral	Treatment and relapse	Protection	**[65]**
Anti-TcdB IgY	TcdB	rTcdB fragment	Chicken	Hamster	Oral *C. difficile* (10^4^ cells)	Oral	Treatment and relapse	Protection	**[65]**
Polyclonal	TcdA and TcdB	rTcdA/B toxoid	Mouse	Hamster	Oral *C. difficile* (10^5^ cells)	I.P.	Prophylactic	Protection	**[66]**
Bovine immune whey	TcdA and TcdB	Culture filtrate	Cow	Hamster	Oral *C. difficile* (10^4^ cells)	Oral	Prophylactic and treatment	Protection	**[67]**
CDA1 IgG	TcdA	Toxoid A	Mouse hybridoma ^3^	Hamster	Oral *C. difficile* spores (140) ^4^	I.P.	Treatment and relapse	Protection	**[7]**
MDX-1388 IgG	TcdB	rTcdB fragment	Mouse hybridoma ^3^	Hamster	Oral *C. difficile* spores (140K) ^4^	I.P.	Treatment and relapse	Protection	**[7]**

^1^. Number of *C. difficile* cells administered was not given. ^2^. *C. difficile* (CD) culture filtrates containing TcdA and TcdB were co-injected into rat ileal loops with anti-toxin bovine Ig. ^3^. Mouse hybridoma cells were generated from HuMAb mice. HuMAb mice are transgenic mice containing human immunoglobulin genes. ^4^. One-hundred and forty (140) *C. difficile* spores were given orally in the treatment model, while 140,000 (140 K) *C. difficile* spores were given orally in the relapse model. I.P. = intraperitoneal. I.V. = intravenous.

**Table 3:** Therapeutic human studies involving *C. difficile* toxin-specific antibodies.

**Antibody**	**Specificity**	**Source**	**Administration Route**	**Number of Treated Patients**	**Treatment Success Rate (%)**	**Ref**
IVIG prep	TcdA	Human	I.V.	5	100	**[53]**
IVIG prep	TcdA and TcdB	Human	I.V.	2	100	**[68]**
IVIG prep	unknown	Human	I.V.	4	100	**[69]**
IVIG prep	unknown	Human	I.V.	5	60	**[70]**
IVIG prep	unknown	Human	I.V.	14	64	**[71]**
IVIG prep	unknown	Human	I.V.	1	100	**[72]**
IVIG prep	unknown	Human	I.V.	18	83	**[76]**
IVIG prep	unknown	Human	I.V.	1	100	**[73]**
IVIG prep	unknown	Human	I.V.	1	100	**[74]**
IVIG prep	unknown	Human	I.V.	2	100	**[75]**
IVIG prep	unknown	Human	I.V.	21	43	**[77]**
IgA	unknown	Human	Oral	1	100	**[78]**
Bovine immune whey	TcdA and TcdB	Cow	Oral	15	93	**[67]**
Bovine immune whey	TcdA and TcdB	Cow	Oral	101	90	**[79]**
Bovine immune whey	TcdA and TcdB	Cow	Oral	20	55	**[80]**
CDA1 IgG	TcdA	Mouse hybridoma ^1^	I.V.	101	93	**[81]**
CDB1 IgG	TcdB	Mouse hybridoma ^1^	I.V.	101	93	**[81]**

^1^ Mouse hybridoma cells were generated from HuMAb mice. HuMAb mice are transgenic mice containing human immunoglobulin genes. IVIG = intravenous immunoglobulin. I.P. = intraperitoneal. I.V. = intravenous.

In another early study examining oral administration of anti-toxin Abs, Lyerly *et al.* [63] showed hamsters could be protected prophylactically from the effects of *C. difficile* with orally administered bovine immunoglobulin G concentrate (BIC), which was generated from the colostrum of cows vaccinated with *C. difficile* culture filtrates. In the post infection model, however, the antibodies had no effect on hamsters. Several years later, Kelly *et al.* [64] produced two bovine IgG preparations by immunizing cattle with *C. difficile* culture filtrates and formalin inactivated TcdA (toxoid A). Both preparations were capable of inhibiting TcdA-induced cytotoxicity in *in vitro* cell assays, as well as inhibiting the enterotoxic effects of TcdA on rat intestinal loops. The study did not assess the efficacy of bovine IgG preparations in either prophylactic or treatment models. 

In a seminal study, Kink and Williams [65] demonstrated that hens immunized with recombinant TcdA and TcdB fragments could yield potent toxin-neutralizing IgY antibodies. As with other studies noted above, only anti-TcdA was required for prophylactic protection. However, when IgY antibodies specific to both toxins were administered orally to hamsters, the effects were profound: hamsters suffering from CDAD were successfully treated and did not show signs of CDAD relapse. This study indicated, for the first time, that neutralization of TcdB was important in treatment of CDAD and prevention of CDAD relapse. Furthermore, this was one of the most successful examples of oral antibody administration, likely due to the robustness of IgY antibodies in withstanding the harsh pH and protease-rich gastrointestinal (GI) tract. Elsewhere, Giannasca *et al.* [66] demonstrated that passive immunization of hamsters with immune hamster sera and polyclonal ascites fluid via the I.P. route resulted in full protection when administered two days before oral *C. difficile* challenge. This study was one of the first to show systemically delivered anti-toxin antibodies could offer mucosal protection from CDAD in hamsters. From this work and that of others, it became obvious that anti-toxin A and B Abs were required for treatment of CDAD. More recently, van Dissel *et al.* [67] showed bovine immune whey preparations containing toxin-specific sIgA and IgG antibodies from immunized cattle were effective at preventing *C. difficile*-induced hamster mortality when administered orally before and after bacterial challenge. Compared to control animals, 80%-90% of hamsters receiving the immune whey survived.

Most of the anti-toxin Ab work reviewed thus far involved antibodies produced from animal sources, but for systemic human therapeutics, antibodies should be humanized or of human origin to reduce potential immunogenicity. Antibody immunogenicity, however, should not be a concern in the oral therapy approach. The first human anti-toxin mAbs specific to TcdA and TcdB were isolated in 2006 and reported by Babcock *et al.* [7]. The group evaluated several antibodies, and found that I.P. administration of their best TcdA-binding candidate (CDA1) combined with their top TcdB-binder (MDX-1388) significantly reduced hamster mortality in the primary CDAD treatment model and CDAD relapse model, relative to either mAb alone. Similar to the most efficacious antibodies reported before, both CDA1 and MDX-1388 recognized the C-terminal host-cell receptor binding domains of TcdA and TcdB, respectively. This work has led to the testing CDA1 and MDX-1388 in the first human clinical trial for the treatment of recurrent CDAD, which is discussed below.

### 4.3. Experimental human studies

A number of studies and case reports have indicated that passive immunotherapy is a successful therapy for human patients suffering from chronic relapsing *C. difficile* infection who did not respond to standard treatment (*i.e.*, antibiotic therapy). In contrast to animal studies where antibodies were delivered orally or systemically, the majority of human studies thus far have used the systemic delivery route. 

The earliest reports of treating relapsing CDAD in humans with antibodies were based on intravenous immunoglobulin (IVIG) therapy (Table 3). IVIG involves injecting high doses of human Ig’s (300-400 mg Ig/kg of body weight) from healthy donors, which are thought to contain TcdA- and TcdB-specific antibodies, into patients suffering from CDAD. The first data showing successful treatment of relapsing CDAD with IVIG was from Leung *et al.* [53] who reported five out of five children were cleared of their symptoms upon receiving 400 mg IVIG/kg. Others have reported similar findings using IVIG therapy with patient survival rates ranging from 60-100%, although these studies lacked control subjects [68,69,70,71,72,73,74,75]. Conversely, a retrospective analysis performed by Juang *et al.* [76] concluded that patients administered IVIG (n = 18) showed no statistical advantages over control groups (n = 18). More recently, Abougergi *et al.* [77] reported 9 of 21 patients (43%) receiving IVIG for severe CDAD survived, indicating one of the highest mortality rates of IVIG thus far.

The first case of orally delivered anti-toxin therapy was reported by Tjellström *et al.* [78] who successfully treated a 3½ year old boy with IgA. Recently, a study by van Dissel *et al.* [67] demonstrated the effectiveness of orally delivered bovine immune whey to CDAD patients. Whey protein enriched in bovine immunoglobulins was prepared from cattle immunized with inactivated *C. difficile* culture filtrates. Of 15 patients receiving the oral immunoglobulin mixture, 14 were completely cured of *C. difficile*-associated diarrhea. The same group then conducted a larger study and found their immune whey treatment successfully prevented CDAD relapse in 98 out of 109 patients [79]. Later, Mattila *et al.* [80] used a similar approach of orally administering bovine immune whey to patients suffering from CDAD and found a 55% success rate, although the clinical trial was prematurely terminated. The discrepancies between the success rates of the first two immune whey studies [67,79] and that conducted by Mattila *et al.* [80] may be due to differences in the immune whey product, patient selection criteria, previous antibiotic usage, and overall study design.

Collectively, these studies illustrated the effectiveness of polyclonal anti-toxin antibody preparations on severe CDAD when administered intravenously or orally to patients. However, a major issue with IVIG therapy or bovine immune concentrates is the quantity, quality and variability of toxin-specific antibodies contained within these preparations. As such, comparing the effectiveness of each of these studies should be treated with caution. 

In a landmark study, Lowy *et al.* [81] recently provided data on the largest clinical trial involving CDAD therapy and the first trial examining the efficacy of human anti-toxin mAbs. The study intravenously administered specific doses of both anti-TcdA mAb CDA1 and anti-TcdB mAb CDB1 or placebo control to 200 patients with recurrent CDAD symptoms. The authors found a significant reduction in CDAD recurrence compared to controls, with only 7% of those receiving the mAb therapy relapsing compared to a 25% relapse rate among patients receiving placebo. 

Finally, comparing the efficacy and success rates of the experimental anti-toxin human studies described here (summarized in Table 3) should be treated with caution since the study design, study endpoints, and success criteria are not uniform. 

### 4.4. Antibody mechanism of action

The process of systemically-administered anti-toxin antibodies neutralizing TcdA and TcdB, which are found primarily in the colon, is not well understood. Two possible explanations have been proposed. First, anti-toxin antibodies administered systemically are thought to migrate to the GI tract through a leaky mucosal barrier [57,82]. With many immune centers located in close proximity to the mucosa barrier, inflamed or disrupted epithelial cells may allow easy access of systemic Abs to the lumen. Alternatively, IgGs may be actively transported from systemic circulation to the lumen via the neonatal IgG Fc receptor (FcRn) which is expressed by colonic epithelial cells [82,83]. Both cases are supported by an increase in the levels of IgG in the stools from infected patients [50]. It is possible that administered Abs may bind and neutralize TcdA/B in the bloodstream, although the principal sight of action is believed to be in the GI tract. Regardless of administration route, systemic or oral, at the molecular level the mechanism of antibody-based toxin-neutralization *in vivo* appears to involve inhibiting the binding of toxin to target cells, a critical first step in the toxins’ mechanism of action (Figure 2).

## 5. Future Perspectives for CDAD Immunotherapy

Many cases documenting the successful treatment of relapsing CDAD with antibody-based reagents have relied on systemically-delivery antibody administration. As mentioned above, these antibodies likely need to reach the GI tract to work effectively. This brings forth the question: Is systemic delivery the most efficacious method for anti-toxin therapy?

Conceivably, oral-administered toxin-neutralizing antibodies would bypass the need for systemically delivered Abs to traverse the mucosal barrier to the GI tract. Currently, there are only a handful of examples in the literature suggesting oral therapy may be effective. This may be largely due to conventional IgGs being sensitive to the extreme pH and protease-rich environment of the stomach and small intestine. Bovine immune Ig preparations [84] and IgA preparations [78] appear to tolerate the GI tract as oral administration has proven effective and functional toxin-specific antibodies have been recovered after GI tract passage. To enhance the efficacy of orally delivered Abs, the exploration of protective antibody formulations and engineered antibodies with robust biophysical properties may be warranted.

### 5.1. Recombinant antibody fragments

Antibody fragments (Figure 3) are smaller versions of parent antibodies (*i.e.*, IgGs) that lack one or more C_H_ domains while retaining antigen binding capacity. In contrast to conventional antibodies (*i.e.*, IgGs) which are produced by traditional immunization approaches, antibody fragments are routinely generated through *in vitro* selection procedures from synthetic/semi-synthetic, naïve, or immune display libraries [48,85]. While both methods are equally important for generating therapeutic antibodies, recombinant antibody (rAb) fragments offer some advantages. One of the main advantages of rAb fragments over conventional antibodies are their amenability to *in vitro* display selection, which circumvents the need for animal immunization and allows for the generation of antibodies against targets that are toxic or infectious to the host. In addition, rAbs can be engineered for greater efficacy, used as scaffolds or building blocks to generate multi-specific or multivalent antibodies, and used as carriers of therapeutic payloads such as radionuclides and toxins. Furthermore, some rAb formats have been shown to bind epitopes that are inaccessible with conventional Abs. The most common rAb formats include Fab (fragment antigen binding), scFv (single chain variable fragments), and single-domain antibodies (see below). Numerous fusion derivatives of these fragments have been engineered [86,87]. 

There are several reports in the literature of potent toxin neutralization with recombinant antibodies, including botulinum toxin, cholera toxin and ricin. With respect to *C. difficile* toxins, there has only been one publication describing the isolation of TcdB binding scFv antibodies [46]. In this work, a hyperimmunized scFv library was constructed and provided a source of toxin binders; however, the work did not progress beyond binding assays. 

### 5.2. Single-domain antibodies

In recent years, smaller antibody fragments have been developed that show considerable promise as human therapeutic and diagnostic agents [48,88,89,90]. Single-domain antibodies (sdAbs) are recombinant, *in vitro* selected fragments and include the V_H_ and V_L_ domains of conventional Igs [91,92,93], the V_H_H domain from *Camelidae* species’ heavy-chain IgGs [94,95,96], and the V_NAR_ domain from cartilaginous shark IgNAR antibodies [97]. The unique feature of these antibodies compared to conventional antibodies is their small size (13-15 kDa) and single-domain nature which consists of only the antigen recognition domain. In addition, sdAbs possess desirable characteristics [98] such as high tissue penetrating properties, high chemical, thermal and proteolytic stability, high level expression in microorganisms, ease of genetic manipulation and library construction and amenability to *in vitro* library screening and selection under harsh conditions such as proteases [99], acidic pH [100] and heat [101]. Because of their small size and the ability to form extended complementarity-determining region 3 (CDR3) loops, sdAbs can access immunosilent cavities in receptors, enzymes, and infectious agents [102,103,104,105] that conventional mAbs cannot access, making them novel and potent inhibitors. 

Several V_H_Hs or ‘nanobodies’ have been isolated against targets relevant to infection and immunity [89]. Many of these V_H_Hs were effective neutralizers of bacterial toxins, viruses, and enzymes, such as: scorpion toxin AahI’ [106]; *E. coli* heat-labile toxin [107]; foot and mouth disease virons [108]; ART2.2, an ecto-enzyme related to ADP-ribosylating bacterial toxins [109]; verotoxin 1 [110]; HIV-1 envelope protein gp 120 [111] and rotovirus [112,113]. With their small size, access to cryptic epitopes, strong binding affinity which can be well into the low picomolar range [114,115,116,117,118,119,120], and intrinsic thermal, chemical and protease stabilities, sdAbs could be promising *C. difficile* toxin-neutralizing agents that may exhibit superior efficacy within the GI tract compared to conventional formats (*i.e.*, IgG). In addition, the efficacy of these compact antibody formats could be further increased by increasing their tolerance to extreme pH and proteolytic degradation through engineering and selection-based approaches. 

## 6. Conclusions

With broad-spectrum antibiotic therapy known to promote *C. difficile* infection and TcdA/B firmly established as the causative agents of *C. difficile*-associated disease, the development of non-antibiotic agents to treat CDAD is of considerable importance. The encouraging results from the first human clinical trial involving two anti-toxin mAbs demonstrated their efficacy in reducing CDAD relapse [81] and further established the importance of anti-toxin neutralizing Abs in controlling CDAD severity. Recently, Demarest *et al.* [121] showed that a panel of mAbs targeting TcdA were potent toxin neutralizers, suggesting oligoclonal mixtures of mAbs or Ab fragments targeting unique epitopes may be superior toxin neutralizers compared to single mAbs targeting a single epitope. Indeed, this has been shown with a panel of anti-botulinum toxin mAbs [122]. 

Pharmacokinetics, pharmacodynamics, affinity, specificity and stability are key determinants of antibody efficacy in CDAD therapy. Higher affinity antibodies, preferably at least with picomolar *K*_D_s, should be aimed for, and with respect to specificity, those capable of blocking toxin binding to the host epithelia or preventing toxin internalization may prove to be most efficacious. Antibody stability, one of the determinants of antibody efficacy in systemic therapy, may be the determining factor of antibody efficacy in the oral therapy approach, given that antibodies have to face the hostile environment of the GI tract with acid-induced denaturing and protease degradation capabilities. Recombinant antibody fragments - in particular single-domain antibodies - lend themselves readily to efficacy improvement with regards to all five of the aforementioned antibody characteristics, thanks to major advances in the past decades within the field of antibody engineering and evolutionary display technologies. Formulation may further protect toxin-specific Abs against the deactivating conditions of the GI tract. Plausibly, probiotic bacteria secreting or displaying recombinant antibody fragments [123,124] specific for TcdA/B could deliver their toxin-neutralizing payloads directly to the lower GI tract, bypassing adverse GI tract conditions and the requirement for purified antibodies altogether. 
